# Differential phase angle spectrum for liquid detection in functionalized surface roughness polymeric electrode

**DOI:** 10.2478/joeb-1024-0019

**Published:** 2024-12-23

**Authors:** John Alexander Gomez-Sanchez, Luciano de Souza Ribero Bueno

**Affiliations:** 1 CalipYuk Enterprises, Rio de Janeiro, Brazil; 2 Agrotechlink, Joinville, Brazil

**Keywords:** dielectric modified surface, biochemical sensor, portable sensor

## Abstract

The conductive polymeric electrodes using 3D printing are an innovative material development with the advantage of the flexibility of integrating isolated polymers with a higher electrical conductivity of carbon-based materials, generating new possibilities in environmental, healthcare, and food monitoring. Based on the morphology, geometric arrangement, and dielectric properties of the composites, the performance of the electrodes is improved. Structural designs are optimized to enhance functionalities such as adhesion, catalytic activity, and the reduction of interface energy. With these concepts, a functionalized carbon-based polymeric electrode was fabricated using 3D printing. The Differential Impedance Spectrum (DIS) was employed to analyze the response of functionalized electrodes to solutions of acetic acid and calcium chloride (*CaCl_2_*). DIS data extract the differential phase angle and indicate the interactions between the functionalized surface with acetic acid and *CaCl_2_* solutions, showing noticeable peaks in three zones: at lower frequencies (< 10 kHz), medium frequencies range of 10 kHz to 100 kHz, and higher frequencies (> 100 kHz). In the three cases analyzed, the *CaCl_2_* solution presented the highest frequency compared with acetic acid. When the sensor was doubled functionalized, the peaks were shifted between the range of 220 kHz to 280 kHz. A conclusion is that the carbon-based polymer electrode not only reduces manufacturing costs but also enables faster functionalization to detect specific chemical compounds in liquids quickly and portable without the need for higher-level equipment. These electrodes could be applied to make measurements in aqueous media such as ponds, pools, lakes, rivers, and oceans to detect contaminants, as well as in human fluids to recognize metabolites in sweat, urine, saliva, and blood.

## Introduction

Plastic is an organic polymeric substance, usually hydrophobic and electrically insulating. When carbon-based functional groups are introduced in a polymer, the surfaces gain some hydrophilicity, and the electrical conductivity improves significantly [1]. The surface functionalization of carbon-based particles such as graphene, carbon nanotubes (CNTs), and activated carbon, are employed as mediating agents in polymeric systems like electrodes, sensors, and catalysts. These particles increase the availability of active sites, alter electromagnetic properties, promote chemical interactions, and improve the efficiency of electron transport [2].

Electrical conductivity and impedance of polymer electrodes need to be adjusted for adequate response. The complexity response of polymers can be enhanced using variations in spatial charge orientation, modifying the electrode geometry, but the polarization may be affected by factors such as internal crystalline structure, combinations with other molecules, and the linear response to an applied electric field [3]. Selecting the appropriate plastic material for polymer electrodes and nanoparticles requires a modelling and simulation, because certain plastics can display varying degrees of polarization when subjected to an electric field [4]

Conductive graphite particles further contribute to the material mechanical flexibility without compromising its conductivity, allowing the composite to be shaped or molded into various geometries. This property is essential for the development of wearable sensors and flexible electronics, where the material must conform to different surfaces [5]. Graphite-polymer electrodes can also be applied in impedance spectroscopy or potentiometric sensors, where surface interactions with target molecules lead to detectable changes in the material electrical properties [6].

3D printing enables precise design and fabrication of customized electrode geometries, this flexibility is particularly advantageous for tailoring sensors to specific detection environments and liquid types. It enables the formulation of intricate electrode designs, such as interdigitated electrodes (IDEs) or complex microstructures, which maximizes surface area for chemical interactions, leading to improved sensor performance. The deliberate introduction of controlled roughness aims to enhance various aspects of electrochemical sensing. Rough surface electrodes increase the contact area for sample interactions, providing several advantages including overall performance, sensitivity, and selectivity [3].

Acrylonitrile Butadiene Styrene (ABS), a polymer filament known for its excellent mechanical properties such as resilience and toughness, is commonly used in 3D printers [7]. When combined with graphite, these polymers maintain or even enhance their structural integrity, making the composite material resistant to various physical stresses, including compression, bending, and stretching [8]. Moreover, 3D printing with ABS and carbon-based materials is relatively low-cost compared to traditional manufacturing methods. It supports scalable production of disposable or reusable sensors, which can be utilized in large-scale environmental monitoring or medical diagnostics, thereby reducing the cost per unit [9].

Additive manufacturing, particularly 3D printing, has increasingly been applied in the sensor development of chemical composes and biological metabolites due to its flexibility and ability to create customized designs. Researchers have produced printed biosensors for detecting glucose levels, pH sensors for measuring the acidity/alkalinity of liquids in both healthcare and environmental monitoring, and lactate biosensors for measuring sweat concentration[10]. Additionally, 3D-printed DNA biosensors have been designed to detect nucleic acids associated with pathogens or genetic markers [11]. Some sensors have been created to detect heavy metals like lead (Pb) or cadmium (Cd) in water, incorporating materials such as Graphene or carbon nanotubes embedded in polymer matrices [12].

Polymeric sensors can be used in impedance measurements utilizing electrochemical impedance spectroscopy (EIS) in both Faradic and non-Faradic measurements. These tests are frequently used in biochemical sensing, medical diagnostics, and environmental monitoring because they provide comprehensive information on electrode behavior and sample based on charge transfer mechanisms, surface responses, and capacitance variations [13]. Conductive polymers like polypyrrole or carbon nanomaterials are often applied to electrode surfaces, enhancing the sensor’s ability to bind metal ions and improving electrochemical detection [14]. Materials such as PLA, Graphene, and gold nanoparticles are also employed in sports physiology and medical diagnostics, particularly in detecting disease biomarkers due to bio-compatibility [15].

The electrical conductivity of ABS-carbon black composites exhibits anisotropic behavior due to the distribution and alignment of carbon black particles within the ABS matrix. This anisotropy results from the differing formation of the carbon black network along the extrusion direction compared to the perpendicular direction. During extrusion or processing, the particles become aligned, creating more robust conductive pathways in specific orientations [16].

The 3D-printed polymer conductive carbon-based sensor can be produced based on computer modeling. Finite element modelling (FEM) helps to support multi-physics simulations, enabling the simultaneous consideration of electromagnetic fields, chemical diffusion, thermal effects, and electrochemical reactions [17]. It also allows for simulation of the integration of conductive materials, such as carbon nanotubes or graphite, and the prediction of key performance metrics such as impedance spectra, capacitance, and surface current density. By fine-tuning the geometry and material properties of polymeric electrodes, FEA facilitates the design of optimized surfaces tailored for specific applications, such as enhancing selectivity in biosensors [18].

A technique known as Differential Impedance Spectra (DIS) is performed by measuring the impedance difference between an active functionalized sensor and a reference sensor across a range of frequencies to analyze their electrical properties. The term "differential" refers to the comparison of impedance measurements taken under different conditions, such as before and after a specific event, chemical reaction, or biological interaction [19]. This approach ensures that the measurements are not affected by temperature fluctuations or changes in the liquid medium, which would similarly impact both sensors. Consequently, this method provides high stability in DIS results over time [20].

The method described in this paper involves measuring the difference in phase angle between the dry sensor and the functionalized sensor for a chemical sample of two solutions, acetic acid and calcium chloride; this approach uses electrical impedance spectroscopy to identify and distinguish chemical substances. The polymeric electrode response shows shifts in phase angle that produce distinct peaks at specific frequencies, enabling the detection of these particular chemical compounds.

## Materials and methods

### Polymeric sensor

The electrode prototype was created using a 3D model printed with an upgraded Ender 3 printer (Creality 3D Technology Co., Ltd). The design was developed in the online 3D software Tinkercad, then parametrized using Ultimaker Cura for print preparation. Print quality was assessed in Ultimaker Cura before the files were uploaded to the printer for production. The nozzle temperature was maintained at 250 °C, while the heated bed was set to 85 °C. The material used for printing was ABS (Acrylonitrile Butadiene Styrene) conductive filament (3D Lab, Brazil), with a volumetric resistivity 10^5^ Ohm/cm and an electrical resistivity surface 10^6^ Ohm.

The sensor has a rectangular shape with two holes at both ends and a set of steps in the middle section that serves as a sample container. The overall dimensions are 40.75 × 11.10 × 5.5 mm (L × W × T), while the dimensions of the sample container are 11.1 × 6.6 mm (L × W). The zoomed area illustrates the specific step heights labeled A, B, C, and D.

#### COMSOL simulation

The polymeric electrode structure, dimensions, and material properties (electrical conductivity and permittivity) of the polymer material and carbon black were included in the AC/DC module in COMSOL 6.0. The additive manufactured model was used as the 3D model, and the materials were defined as ABS Conductive, with properties supplied by the manufacturer. The polarization norm gives insight into the strength and direction of dipole moments or charge distributions in the polymeric material.

#### Experimental setup

The experiments were conducted in three sets for each solution, first without functionalization (WF), second with the electrode surface functionalized for 30 minutes with 0.25 ml of oil-based biotin (BF), and the third using a hydrated electrode (first step) functionalized with oilbased biotin. The following sequences were used:

- WF+Acetic: Dry Electrode + 0.25 ml of Acetic.
- WF+*CaCl_2_*: Dry Electrode + 0.25 ml of *CaCl_2_*.
- BF+Acetic: Dry Electrode + 0.25 ml of Biotin + Clean + 0.25 ml of Acetic.
- BF+*CaCl_2_*: Dry Electrode + 0.25 ml of Biotin + Clean + 0.25 ml of *CaCl_2_*.
- WF+Acetic+Biotin+Acetic: Dry Electrode + 0.25 ml of Acetic + Clean + 0.25 ml of Biotin + Clean + 0.25 ml of Acetic.
- WF+*CaCl_2_*+Biotin+*CaCl_2_*: Dry Electrode + 0.25 ml of *CaCl_2_* + Clean + 0.25 ml of Biotin + Clean + 0.25 ml of *CaCl_2_*.

#### Impedance measurements

A Zurich Instruments MFLI impedance analyzer was connected to a circuit on a prototyping board that includes a polymeric electrode. The impedance was measured across frequencies ranging from 10 Hz to 1 MHz. The Zurich impedance analyzer uses a two-terminal (bipolar) configuration: one terminal (LCUR + LPOT) and the second terminal (HCUR + HPOT). The impedance spectrum was repeated five times at each frequency point, allowing noise averaging and improving signal quality.

#### Differential phase angle spectrum

Using the impedance phase angle data collected from the Zurich impedance analyzer, the differential phase angle spectrum was calculated as the absolute value of the difference between the phase angles of the functionalized sensor and the dry sensor for the same frequency, multiplied by 100.

1 $$\text{Δ}\phi (f)=\left|\left(\phi_{\text{functionalized }}(f)-\phi_{\text{dry }}(f)\right)\right|\times 100$$

Where:

- △*ϕ*: represents the differential phase angle.
- *ϕ*_functionalized_: represents the phase angle of the functionalized sensor.
- *ϕ*_dry_: represents the phase angle of the dry sensor.

#### Ethical approval

The conducted research is not related to either human or animal use.

### Results

Figure 4 shows a differential phase angle spectrum in a wide frequency range from 10 Hz to 1 MHz. The x-axis represents the frequency in a logarithmic scale, spanning from 10 Hz to 1 MHz, and the y-axis represents the phase shift (differential Phase Angle). Each line represents a different experimental condition that affects the sensor response:

- Blue dashed line (WF+Acetic): This curve has a noticeable peak between 1 kHz and 10 kHz (around 3-5 kHz), with a maximum differential phase of 10.5±0.11, presents another peak between 60 kHz and 300 kHz with a value of 3.8±0.01.
- Blue triangle Line (WF+*CaCl_2_*): This curve has a noticeable peak between 2 kHz and 20 kHz (around 5-7 kHz), with a maximum differential phase of 13.5±0.05, presents another peak between 100 kHz and 500 kHz with a value of 7.8±0.03.
- Orange dashed line (BF+Acetic): This curve peaks around 30 kHz and shows a differential phase response of around 13.2±0.07.
- Orange circle line (BF+*CaCl_2_*): this curve follows a pattern similar to the orange dashed line but shifts slightly towards a higher frequency range, reaching a peak at 247.7 kHz with 14.3±0.09 of differential phase.
- Green dashed line (WF+Acetic+Biotin+Acetic): This curve has a broader peak around 200 kHz, with a differential phase above 11.1±0.06.
- Green square line (WF+*CaCl_2_*+Biotin+*CaCl_2_*): This curve shows a prominent peak at 278.2 kHz and exhibits the highest differential phase response of 16.5±0.13.

Figure 5 shows the phase and impedance spectra of four dry sensors (10 Hz to 1 MHz), all sensors exhibit the usual capacitive behavior of decreasing impedance as frequency increases. The impedance values vary slightly at low frequencies (less than 100 Hz), but they converge rapidly as the frequency increases. The impedance is consistently the lowest over the frequency range for Dry sensor 1 (solid green line) and somewhat higher for Dry sensor 2 (blue dotted line). The phase spectra show that the polymeric electrodes exhibit frequencydependent capacitive behavior, with a very low capacitive contribution and stronger capacitive effects at higher frequencies. Among the sensors, Dry sensor 2 appears to have the highest capacitive contribution, while Dry sensors 1 and 3 have the lowest. The observed trends align with typical behavior in systems where capacitors dominate the impedance at higher frequencies, while resistive components dominate at lower frequencies. The phase angle varies minimally but highlights subtle differences among the sensors, with Dry sensor 2 deviating slightly more than others since 10 Hz.

**Figure 1:**
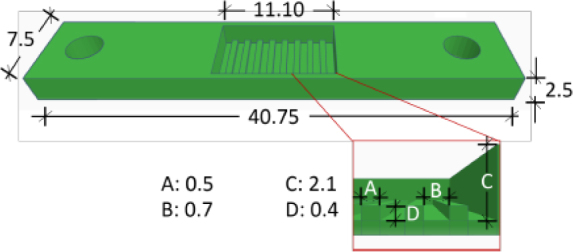
Polymeric biosensor dimensions in millimeters. A: Distance between the steps, B: Height of one step, C: Overall depth of the stepped section, D: Internal step of the box.

**Figure 2:**
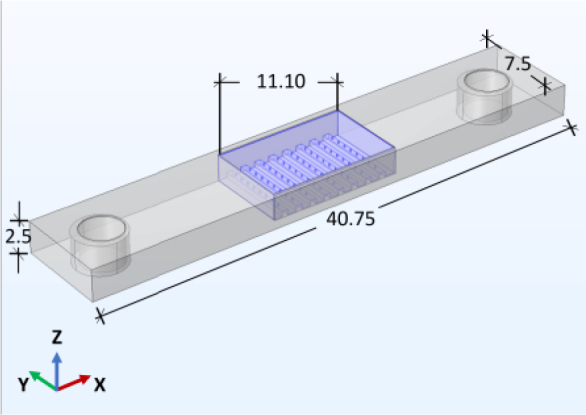
3D model for simulations in COMSOL 6.0

**Figure 3:**
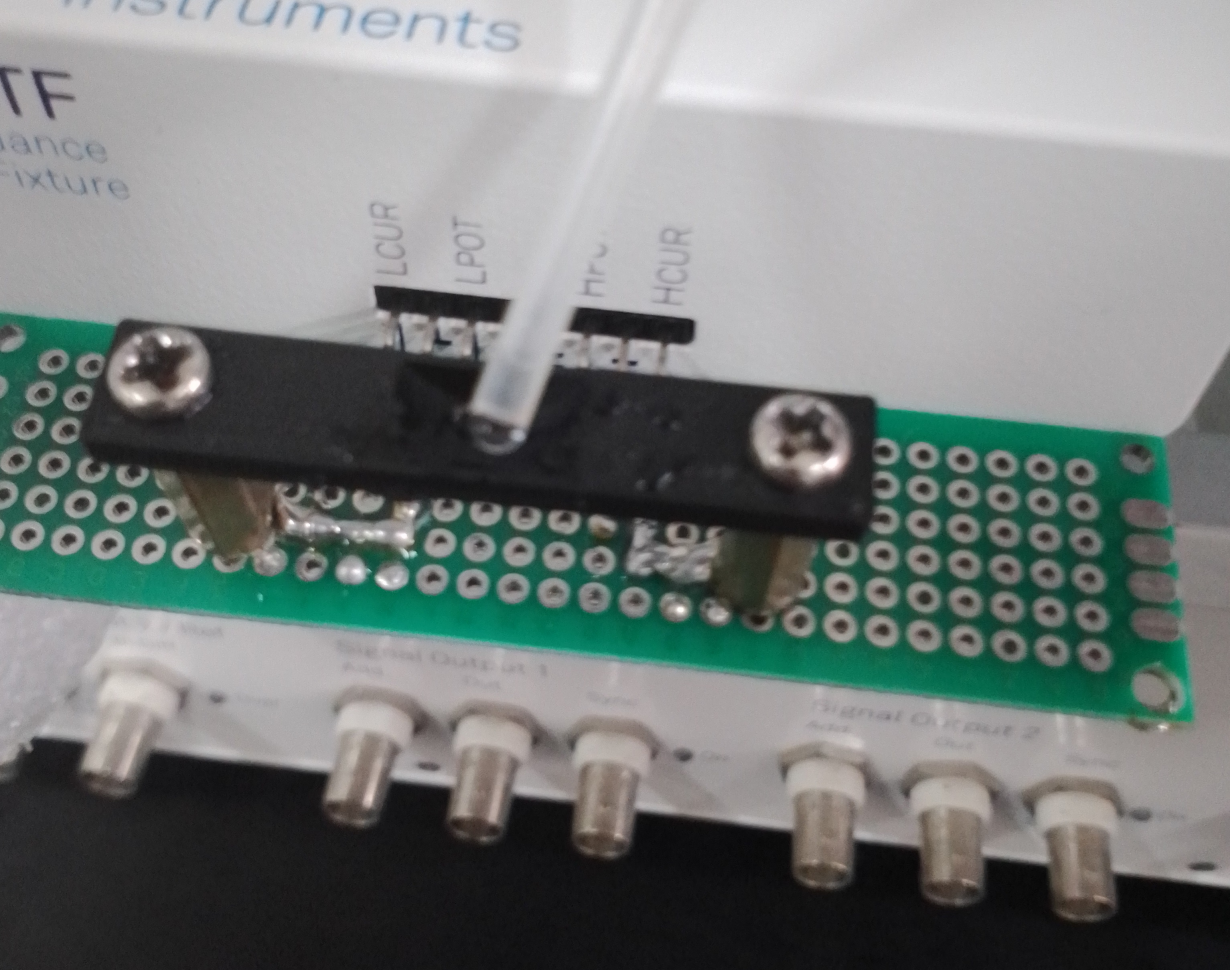
Setup of impedance measurements using Zurich Instruments MFLI

**Figure 4:**
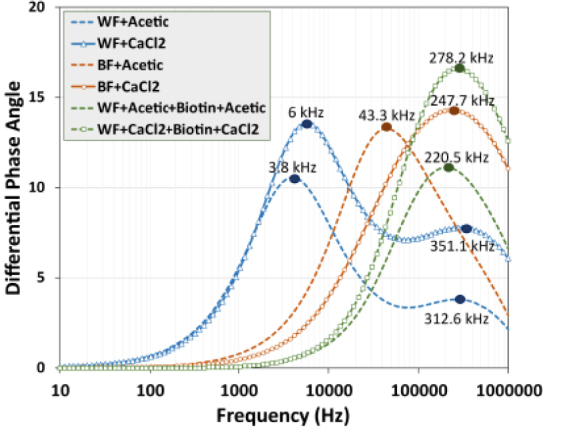
Differential angle phase spectrum for the functionalized electrode surface in the presence of acetic acid and *CaCl_2_*. Blue dashed line (WF + Acetic), blue triangle line (WF + *CaCl_2_*), orange dashed line (BF + Acetic), circle line (BF + *CaCl_2_*), green dashed line (WF + Acetic + Biotin), and green square line (WF + *CaCl_2_* + Biotin + *CaCl_2_*).

**Figure 5:**
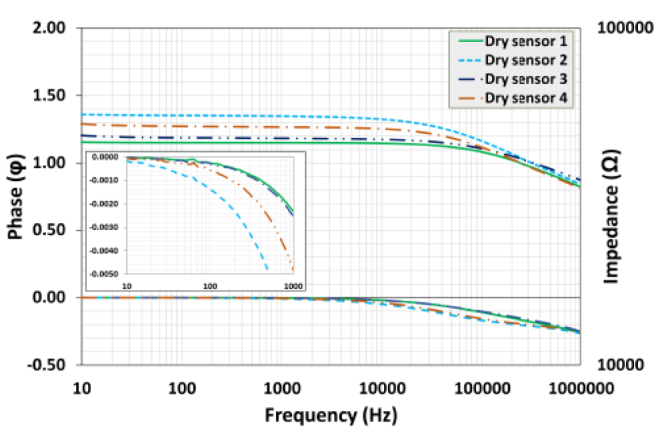
Frequency-dependent impedance and phase response of dry polymeric electrodes: Analysis of dry sensors 1 (green), 2 (blue dashed), 3 (navy blue), and 4 (orange dashed-dot)

**Figure 6:**
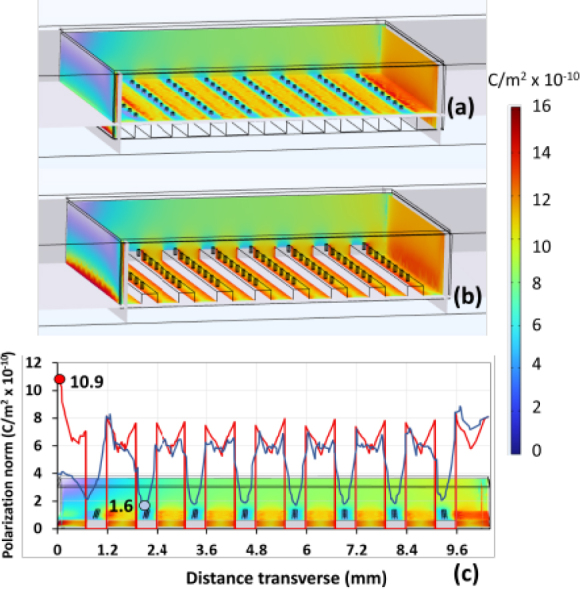
COMSOL simulation of surface polarization on the steps in the sample box. Blue line: Top surface, Red line: Bottom surface. Electric potential distribution (a) top step and (b) bottom step ;(c) Polarization dynamics as a function of position

Figure 6 presents a COMSOL simulation of the polarization norm for the top and bottom surfaces of the polymeric electrode step. The color scale on the right represents the magnitude of polarization, with values ranging from 1.6 to 10.9 C/m^2^ × 10^−10^. On the top surface of the step, visible zones of high polarization appear near the borders, indicating localized areas of strong electric response (blue line). In contrast, the bottom cross section of the sample shows a clear polarization gradient, although the distribution of highpolarization zones differs slightly compared to the top surface (red line). The arrangement of these highpolarization regions along the electrode step corresponds to the applied electric field, with more intense polarization occurring at the edges. Additionally, the transverse walls of the sample box display two distinct behaviors: the left side exhibits a steeper gradient compared to the right side.

## Discussion

### Frequency response

The blue curve in Figure 4 represents the sensor response without functionalization (hydrophobic) for acetic acid and *CaCl_2_*. In low frequencies around 3 to 7 kHz, this higher peak response appears as a result of the ionic interactions of each solution. The acetic acid solution interacts less with the electrode surface than calcium chloride. In the frequency range of 10 and 100 kHz, the behavior is dominated by a dipole orientation with fewer ionic movements [21], decreasing the ionic polarization and the delta phase. A lower peak appears for acetic acid and calcium chloride solutions in frequencies above 300 kHz, this behavior is compared with dual frequency ionic relaxation in ionic liquids [22, 23].

The orange curves show the response of acetic acid and *CaCl_2_* in the mid-frequency range (30 kHz to 400 kHz) of the biotin-functionalized sensor (hydrophilic). The reaction of acetic acid is caused by the interaction of negatively charged acetate ions (*CH3COO*^–^) with positively polarized regions functionalized electrode surface; the higher peak appears at 43.3 kHz. The electrostatic attraction between the functionalized electrode surface occurs between a biotin carboxyl (-COOH) group and the positively charged ions of calcium chloride solution, these electrostatic interactions between *Ca^2+^* and biotin are relatively weak and transient and present a peak in 247.7 kHz. The difference in frequency response is due to the capacitive interface created for the oiled-based biotin over the surface of the conductive polymeric electrode. Calcium ions had a higher charge and a smaller effective ionic radius, leading to more pronounced dielectric relaxation effects. The phase angle peak for calcium ions typically shifts to a slightly higher frequency compared with acetate due to their slower mobility [24].

The green curves show the frequency response of the polymeric conductive electrode functionalization sequence with two aqueous solutions (acetic acid and calcium chloride) and oil-based biotin. The curves exhibit strong peaks in frequencies between 220 and 280 kHz for the sample solution. This result suggests a significant electrode functionalization between the aqueous solution and oiledbased biotin for affinity interaction and sensibility of each sample of acetic acid and calcium chloride.

### Electric field

The mixture of ABS and carbon black maintains a predominant hydrophobic surface with a conductive and polarizable enhanced matrix; the surface is heterogeneous and causes interactions with polar and nonpolar solutions, these interactions are due to a slightly negative surface that attracts ions by electrostatic forces. The results of the differential impedance spectrum (differential phase angle vs. Frequency) in Figure 4 show how the functionalization of the electrode with different materials affects the frequency impedance response.

The electric field distribution in electrodes made from polymers and nanoparticles has been previously studied, including geometrical surface modifications [3]. This occurs as a result of nanoscale attraction, functionalization enhances the quality of interactions in carbon-based composites [2]. The carbon-black particles enhance the conductivity and polarizability around the corners. The polarization gradient indicates several material responses, possibly due to the non-uniform distribution of carbon particles. This could suggest anisotropy in the material properties with a patterned distribution of carbon particles. The selected pattern surface shows alternating regions of high and low polarization on the electrode (Figure 6); this pattern configuration creates zones of local electric field gradients that increase the frequency response of the material to external electric fields and charge carriers.

The variation between figure 6 (a) and (b) indicates different cross-sections or perspectives of the same structure, both confirming that the polarization is not uniform but rather highly dependent on the material composition and geometry. The alternating regions of high and low polarization suggest that the material composition or geometry optimized the electric properties as conductivity and surface permittivity. The high polarization zones suggest efficient charge accumulation or dipole alignment in certain regions.

## Conclusion

The research highlights the potential of combining advanced materials and 3D printing in sensor technology. Additive manufacturing allows for precise customization of electrode geometries, facilitating designs that maximize surface area and improve detection sensitivity. The functionalized carbon-polymer electrodes provide a scalable and flexible solution for detecting chemical substances in various liquid media, ranging from environmental water sources to human fluids. The study demonstrated distinct impedance responses for chemical samples, showing the method’s efficacy in analyzing and identifying liquids across different frequency bands. Future directions could include further refinement of sensor sensitivity, expansion to broader chemical analyses, and integration with automated data systems for real-time monitoring. This technology offers a cost-effective, portable, and rapid alternative for chemical detection, suitable for applications in environmental monitoring and medical diagnostics as wearable devices.

## References

[1] Briesemeister M, Gómez-Sánchez JA, Bertemes-Filho P, Pezzin SH. (2024). PVC/CNT Electrospun Composites: Morphology and Thermal and Impedance Behavior. Polymers.

[2] deOliveira TC, Ferreira F, deMenezes BRC, Silva DM da, Santos AS, Kawachi EY, Simonetti EAN, Cividanes LS. (2021). Engineering the surface of carbon-based nanomaterials for dispersion control in organic solvents or polymer matrices. Surfaces and Interfaces.

[3] Gomez-Sanchez JA, Bueno LSR, Bertemes-Filho P. (2024). Evaluation of electric field in polymeric electrodes geometries for liquid biosensing applications using COMSOL multiphysics. Sensing and Bio-Sensing Research.

[4] LaFreniere JMJ, Roberge EJ, Halpern JM. (2020). Review—Reorientation of Polymers in an Applied Electric Field for Electrochemical Sensors. Journal of The Electrochemical Society.

[5] Htwe YZN, Mariatti M. (2022). Printed graphene and hybrid conductive inks for flexible, stretchable, and wearable electronics: Progress, opportunities, and challenges. Journal of Science: Advanced Materials and Devices.

[6] Pividori MI, Merkoçi A, Alegret S. (2003). Graphite-epoxy composites as a new transducing material for electrochemical genosensing. Biosensors and Bioelectronics.

[7] Mishra V, Ror CHK, Negi S, Kar S, Borah LN. (2023). 3D printing with recycled ABS resin: Effect of blending and printing temperature. Materials Chemistry and Physics.

[8] Pandey AK, Kumar R, Kachhavaha VS, Kar KK. (2016). Mechanical and thermal behaviours of graphite flake-reinforced acrylonitrile–butadiene–styrene composites and their correlation with entanglement density, adhesion, reinforcement and C factor. RCS Advances.

[9] Dananjaya SAV, Chevali VS, Dear JP, Potluri P, Abeykoon C. (2024). 3D printing of biodegradable polymers and their composites – Current state-of-the-art, properties, applications, and machine learning for potential future applications. Progress in Materials Science.

[10] Min J, Tu J, Xu C, Lukas H, Shin S, Yang Y, Solomon SA, Mukasa D, Gao W. (2023). Skin-Interfaced Wearable Sweat Sensors for Precision Medicine. Chemical Reviews.

[11] Erdem A, Yildiz E, Senturk H, Maral (2023). Implementation of 3D printing technologies to electrochemical and optical biosensors developed for biomedical and pharmaceutical analysis. Journal of Pharmaceutical and Biomedical Analysis.

[12] Khalil A, Hashaikeh R, Hilal N. (2021). 3D printed zeolite-Y for removing heavy metals from water. Journal of Water Process Engineering.

[13] Muñoz J, Montes R, Baeza M. (2017). ETrends in electrochemical impedance spectroscopy involving nanocomposite transducers: Characterization, architecture surface and bio-sensing. TrAC Trends in Analytical Chemistry.

[14] Wilczewska P, Breczko J, Bobrowska DM, WysockaŻołopa M, Goclon J, Basa A, Winkler K. (2022). Enhancement of polypyrrole electrochemical performance with graphene quantum dots in polypyrrole nanoparticle/graphene quantum dot composites. Journal of Electroanalytical Chemistry.

[15] Zhou W, Cheng F, Cai C, Fu Y. (2023). Bioinspired dry-state polylactic acid adhesives-based wearable sensor with reversible adhesive performance in harsh environments via building hierarchical liquid metal bead structure. Composites Science and Technology.

[16] Ecco LG, Dul S, Pereira-Schmitz D, deOliveira-Barra GM, Guenther-Soares B, Fambri L, Pegoretti A. (2018). Rapid Prototyping of Efficient Electromagnetic Interference Shielding Polymer Composites via Fused Deposition Modeling. Applied Sciences.

[17] Monica PR, Chaubey N, Sreedevi VT. (2016). An optimized geometry-physics based compact model of CNTFET. Material Today: Proceedings.

[18] Hossain J, Tabatabaei BT, Kiki M, Choi JW. (2024). Additive Manufacturing of Sensors: A Comprehensive Review. International Journal of Precision Engineering and Manufacturing-Green Technology.

[19] Gasser A, Eveness J, Kiely J, Attwood D, Luxton R. (2020). A non-contact impedimetric biosensing system for classification of toxins associated with cytotoxicity testing. Bioelectrochemistry.

[20] Piedimonte P, Sola L, Cretich M, Gori A, Chiari M, Marchisio E, Borga P, Bertacco R, Melloni A, Ferrari G, Sampietro M. (2022). Differential Impedance Sensing platform for high selectivity antibody detection down to few counts: A case study on Dengue Virus. Biosensors and Bioelectronics.

[21] Arya A, Sharma A. (2019). Temperature and Salt-Dependent Dielectric Properties of Blend Solid Polymer Electrolyte Complexed with LiBOB. Macromolecular Research.

[22] Buehler M, Cobos D, Dunne K. (2011). Dielectric constant and osmotic potential from ion-dipole polarization measurements of KCl-and NaCl-doped aqueous solutions. Proc. of the 9th Int. Conf. on Electromagnetic Wave Interaction with Water and Moist Substances (ISEMA 2011) (Missouri, June 2011).

[23] Rivera A, Rössler EA. (2006). Evidence of secondary relaxations in the dielectric spectra of ionic liquids. Physical Review B.

[24] Aswathy PK, Ganga R, Rajendran DN. (2022). Impact of A-site calcium on structural and electrical properties of samarium cobaltite perovskites. Solid State Communications.

